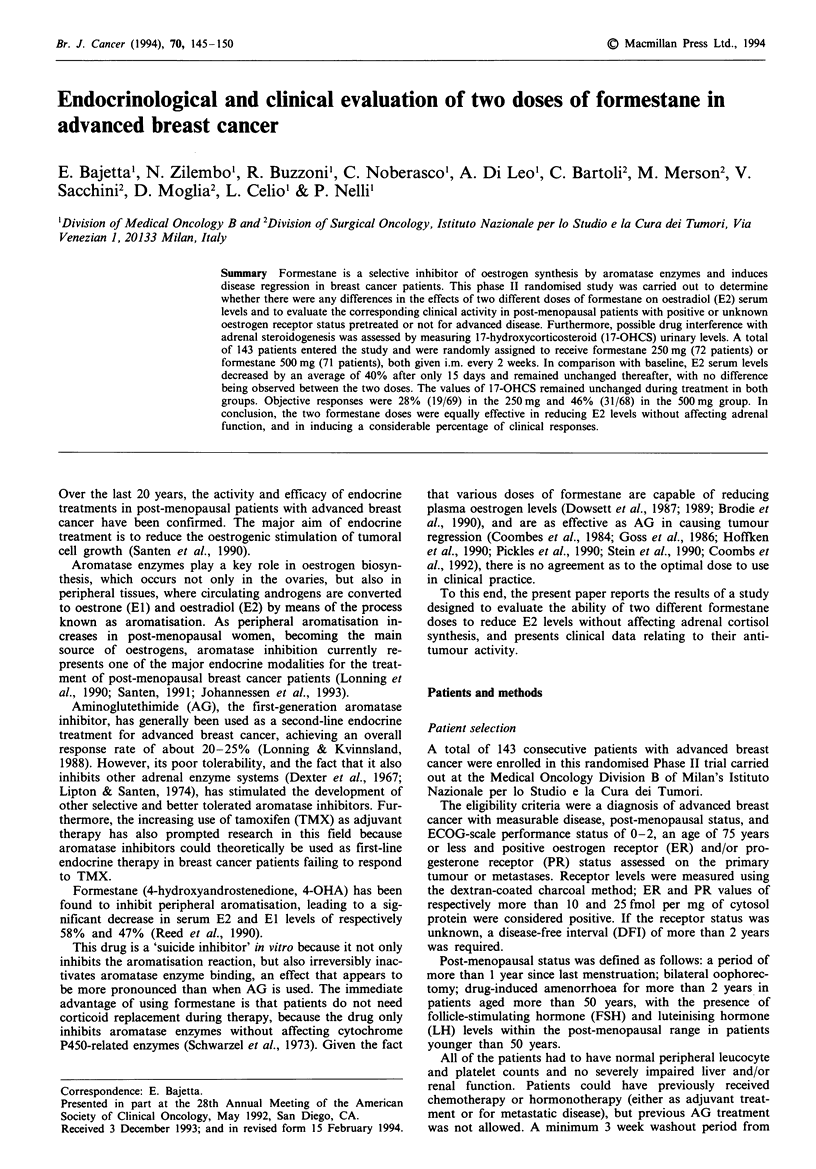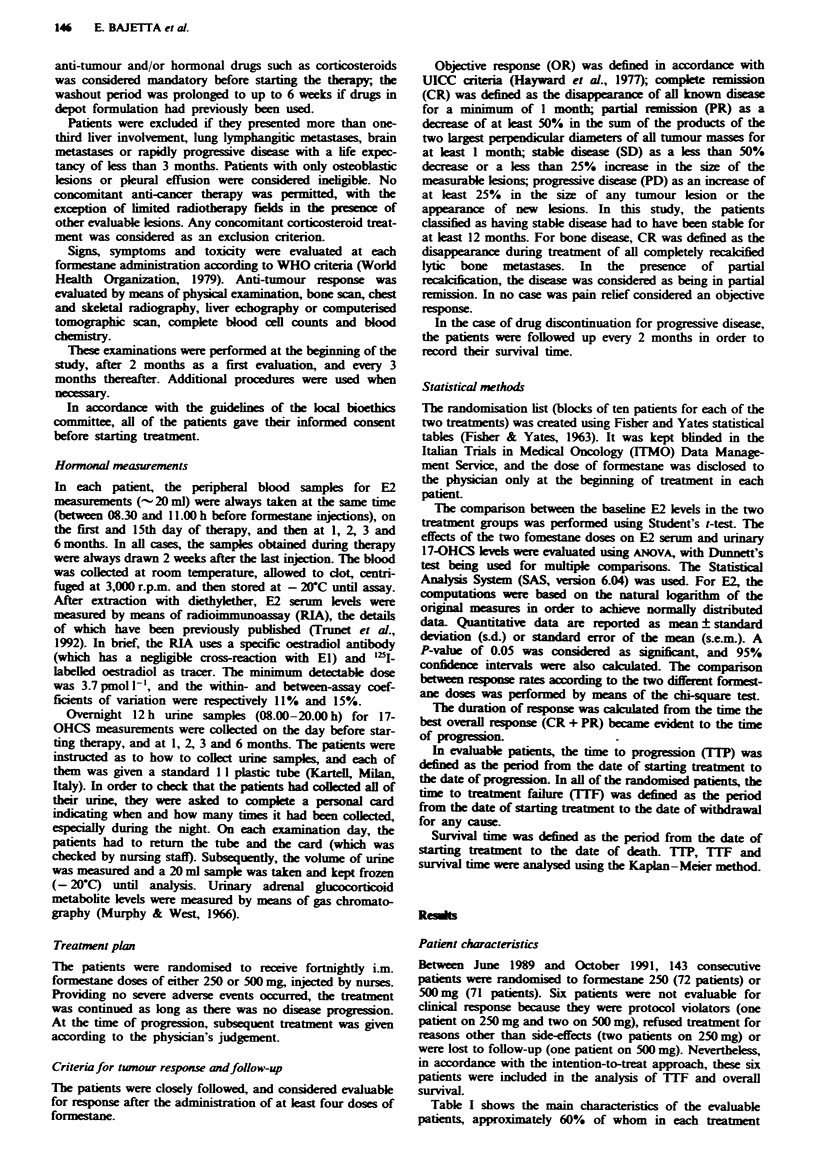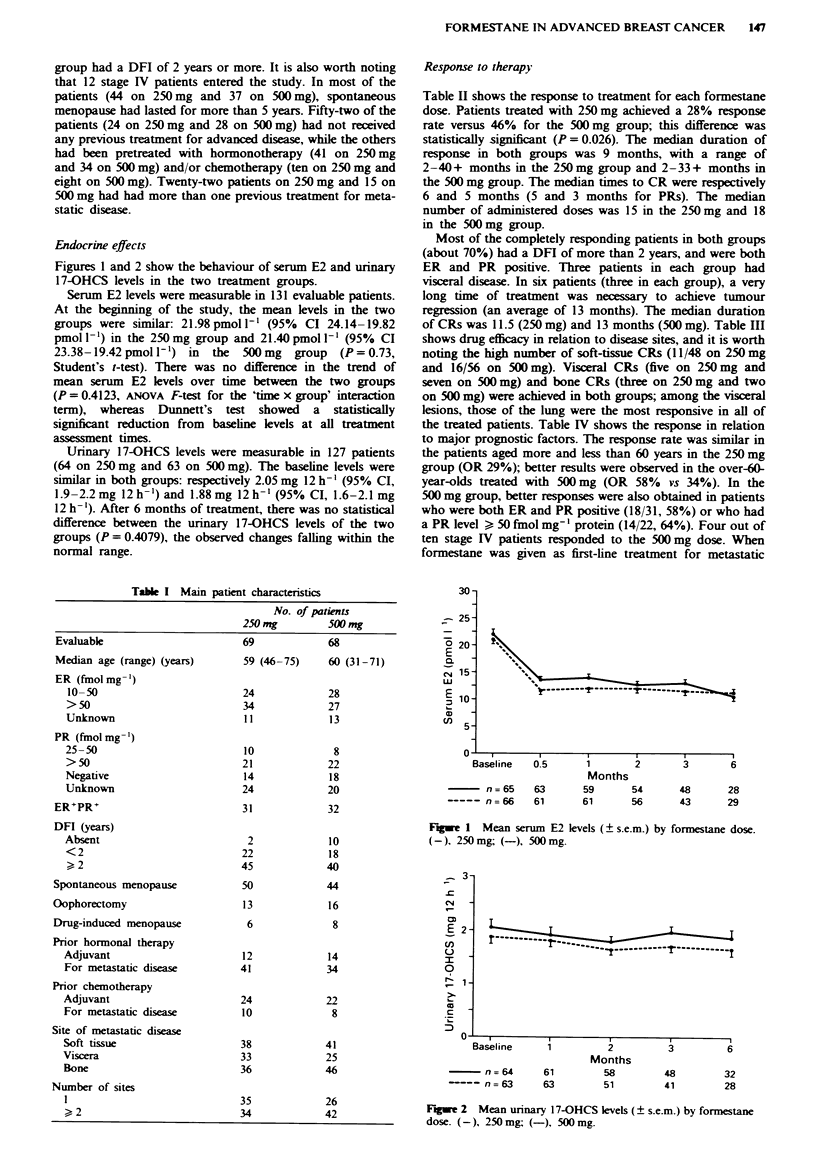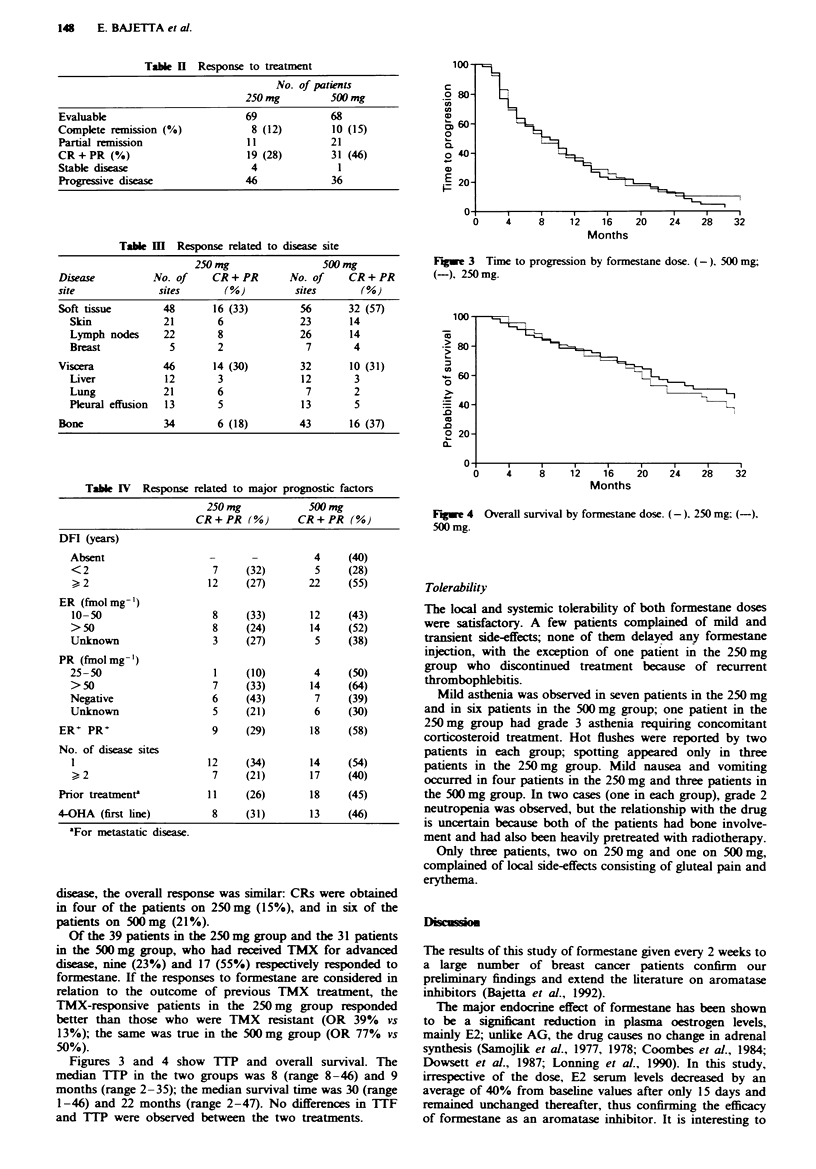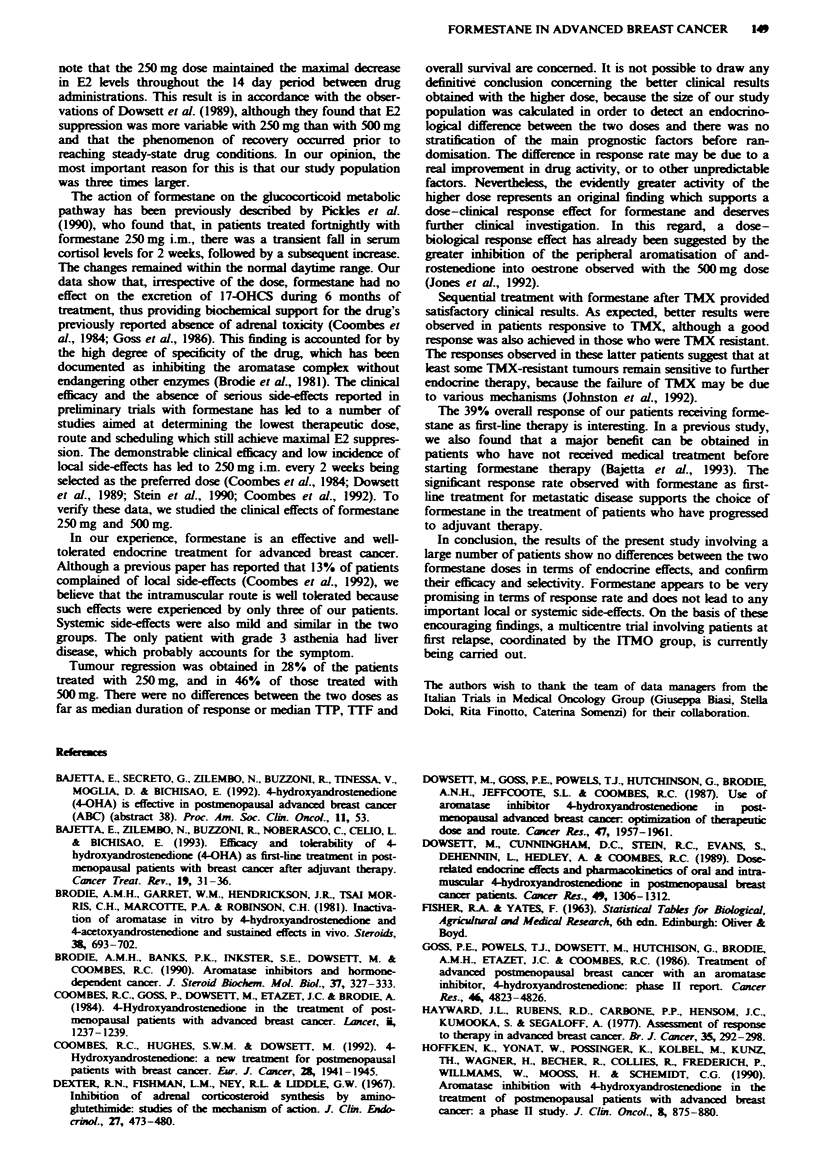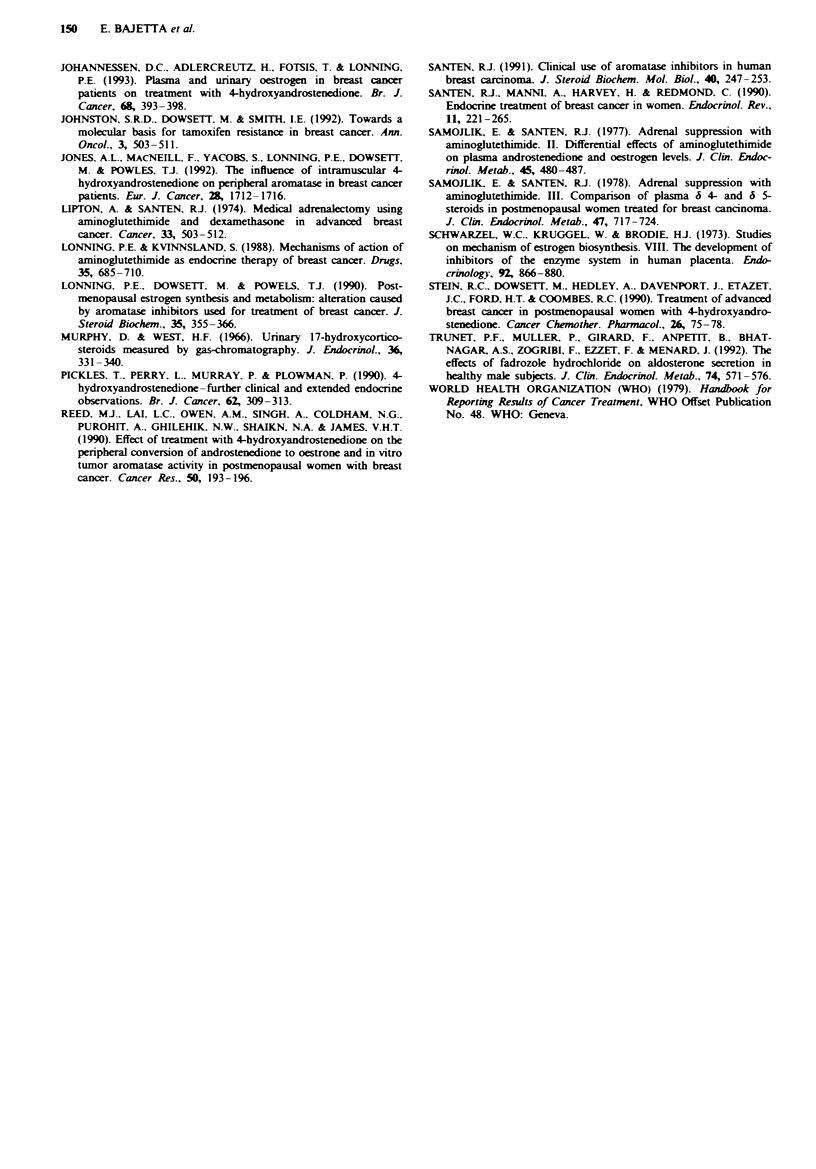# Endocrinological and clinical evaluation of two doses of formestane in advanced breast cancer.

**DOI:** 10.1038/bjc.1994.265

**Published:** 1994-07

**Authors:** E. Bajetta, N. Zilembo, R. Buzzoni, C. Noberasco, A. Di Leo, C. Bartoli, M. Merson, V. Sacchini, D. Moglia, L. Celio

**Affiliations:** Division of Medical Oncology B, Istituto Nazionale per lo Studio e la Cura dei Tumori, Milan, Italy.

## Abstract

Formestane is a selective inhibitor of oestrogen synthesis by aromatase enzymes and induces disease regression in breast cancer patients. This phase II randomised study was carried out to determine whether there were any differences in the effects of two different doses of formestane on oestradiol (E2) serum levels and to evaluate the corresponding clinical activity in post-menopausal patients with positive or unknown oestrogen receptor status pretreated or not for advanced disease. Furthermore, possible drug interference with adrenal steroidogenesis was assessed by measuring 17-hydroxycorticosteroid (17-OHCS) urinary levels. A total of 143 patients entered the study and were randomly assigned to receive formestane 250 mg (72 patients) or formestane 500 mg (71 patients), both given i.m. every 2 weeks. In comparison with baseline, E2 serum levels decreased by an average of 40% after only 15 days and remained unchanged thereafter, with no difference being observed between the two doses. The values of 17-OHCS remained unchanged during treatment in both groups. Objective responses were 28% (19/69) in the 250 mg and 46% (31/68) in the 500 mg group. In conclusion, the two formestane doses were equally effective in reducing E2 levels without affecting adrenal function, and in inducing a considerable percentage of clinical responses.


Br. J. Cancer (1994), 70, 145–150 © Macmillan Press Ltd., 1994

### Endocrinological and clinical evaluation of two doses of formestane in advanced breast cancer

E. Bajetta[1], N. Zilembo[1], R. Buzzoni[1], C. Noberasco[1], A. Di Leo[1], C. Bartoli[2], M. Merson[2], V. Sacchini[2], D. Moglia[2], L. Celio[1] & P. Nelli[1]

[1]*Division of Medical Oncology B and* [2]*Division of Surgical Oncology, Istituto Nazionale per lo Studio e la Cura dei Tumori, Via Venezian 1, 20133 Milan, Italy*

**Summary** Formestane is a selective inhibitor of oestrogen synthesis by aromatase enzymes and induces disease regression in breast cancer patients. This phase II randomised study was carried out to determine whether there were any differences in the effects of two different doses of formestane on oestradiol (E2) serum levels and to evaluate the corresponding clinical activity in post-menopausal patients with positive or unknown oestrogen receptor status pretreated or not for advanced disease. Furthermore, possible drug interference with adrenal steroidogenesis was assessed by measuring 17-hydroxycorticosteroid (17-OHCS) urinary levels. A total of 143 patients entered the study and were randomly assigned to receive formestane 250 mg (72 patients) or formestane 500 mg (71 patients), both given i.m. every 2 weeks. In comparison with baseline, E2 serum levels decreased by an average of 40% after only 15 days and remained unchanged thereafter, with no difference being observed between the two doses. The values of 17-OHCS remained unchanged during treatment in both groups. Objective responses were 28% (19/69) in the 250 mg and 46% (31/68) in the 500 mg group. In conclusion, the two formestane doses were equally effective in reducing E2 levels without affecting adrenal function, and in inducing a considerable percentage of clinical responses.

Over the last 20 years, the activity and efficacy of endocrine treatments in post-menopausal patients with advanced breast cancer have been confirmed. The major aim of endocrine treatment is to reduce the oestrogenic stimulation of tumoral cell growth (Santen *et al.*, 1990).

Aromatase enzymes play a key role in oestrogen biosynthesis, which occurs not only in the ovaries, but also in peripheral tissues, where circulating androgens are converted to oestrone (E1) and oestradiol (E2) by means of the process known as aromatisation. As peripheral aromatisation increases in post-menopausal women, becoming the main source of oestrogens, aromatase inhibition currently represents one of the major endocrine modalities for the treatment of post-menopausal breast cancer patients (Lonning *et al.*, 1990; Santen, 1991; Johannessen *et al.*, 1993).

Aminoglutethimide (AG), the first-generation aromatase inhibitor, has generally been used as a second-line endocrine treatment for advanced breast cancer, achieving an overall response rate of about 20–25% (Lonning & Kvinnsland, 1988). However, its poor tolerability, and the fact that it also inhibits other adrenal enzyme systems (Dexter *et al.*, 1967; Lipton & Santen, 1974), has stimulated the development of other selective and better tolerated aromatase inhibitors. Furthermore, the increasing use of tamoxifen (TMX) as adjuvant therapy has also prompted research in this field because aromatase inhibitors could theoretically be used as first-line endocrine therapy in breast cancer patients failing to respond to TMX.

Formestane (4-hydroxyandrostenedione, 4-OHA) has been found to inhibit peripheral aromatisation, leading to a significant decrease in serum E2 and E1 levels of respectively 58% and 47% (Reed *et al.*, 1990).

This drug is a 'suicide inhibitor' *in vitro* because it not only inhibits the aromatisation reaction, but also irreversibly inactivates aromatase enzyme binding, an effect that appears to be more pronounced than when AG is used. The immediate advantage of using formestane is that patients do not need corticoid replacement during therapy, because the drug only inhibits aromatase enzymes without affecting cytochrome P450-related enzymes (Schwarzel *et al.*, 1973). Given the fact that various doses of formestane are capable of reducing plasma oestrogen levels (Dowsett *et al.*, 1987; 1989; Brodie *et al.*, 1990), and are as effective as AG in causing tumour regression (Coombes *et al.*, 1984; Goss *et al.*, 1986; Hoffken *et al.*, 1990; Pickles *et al.*, 1990; Stein *et al.*, 1990; Coombs *et al.*, 1992), there is no agreement as to the optimal dose to use in clinical practice.

To this end, the present paper reports the results of a study designed to evaluate the ability of two different formestane doses to reduce E2 levels without affecting adrenal cortisol synthesis, and presents clinical data relating to their antitumour activity.

### Patients and methods

### *Patient selection*

A total of 143 consecutive patients with advanced breast cancer were enrolled in this randomised Phase II trial carried out at the Medical Oncology Division B of Milan's Istituto Nazionale per lo Studio e la Cura dei Tumori.

The eligibility criteria were a diagnosis of advanced breast cancer with measurable disease, post-menopausal status, and ECOG-scale performance status of 0–2, an age of 75 years or less and positive oestrogen receptor (ER) and/or progesterone receptor (PR) status assessed on the primary tumour or metastases. Receptor levels were measured using the dextran-coated charcoal method; ER and PR values of respectively more than 10 and 25 fmol per mg of cytosol protein were considered positive. If the receptor status was unknown, a disease-free interval (DFI) of more than 2 years was required.

Post-menopausal status was defined as follows: a period of more than 1 year since last menstruation; bilateral oophorectomy; drug-induced amenorrhoea for more than 2 years in patients aged more than 50 years, with the presence of follicle-stimulating hormone (FSH) and luteinising hormone (LH) levels within the post-menopausal range in patients younger than 50 years.

All of the patients had to have normal peripheral leucocyte and platelet counts and no severely impaired liver and/or renal function. Patients could have previously received chemotherapy or hormonotherapy (either as adjuvant treatment or for metastatic disease), but previous AG treatment was not allowed. A minimum 3 week washout period from

Correspondence: E. Bajetta.
Presented in part at the 28th Annual Meeting of the American Society of Clinical Oncology, May 1992, San Diego, CA.
Received 3 December 1993; and in revised form 15 February 1994.

anti-tumour and/or hormonal drugs such as corticosteroids was considered mandatory before starting the therapy; the washout period was prolonged to up to 6 weeks if drugs in depot formulation had previously been used.

Patients were excluded if they presented more than one-third liver involvement, lung lymphangitic metastases, brain metastases or rapidly progressive disease with a life expectancy of less than 3 months. Patients with only osteoblastic lesions or pleural effusion were considered ineligible. No concomitant anti-cancer therapy was permitted, with the exception of limited radiotherapy fields in the presence of other evaluable lesions. Any concomitant corticosteroid treatment was considered as an exclusion criterion.

Signs, symptoms and toxicity were evaluated at each formestane administration according to WHO criteria (World Health Organization, 1979). Anti-tumour response was evaluated by means of physical examination, bone scan, chest and skeletal radiography, liver echography or computerised tomographic scan, complete blood cell counts and blood chemistry.

These examinations were performed at the beginning of the study, after 2 months as a first evaluation, and every 3 months thereafter. Additional procedures were used when necessary.

In accordance with the guidelines of the local bioethics committee, all of the patients gave their informed consent before starting treatment.

### Hormonal measurements

In each patient, the peripheral blood samples for E2 measurements (~20 ml) were always taken at the same time (between 08.30 and 11.00 h before formestane injections), on the first and 15th day of therapy, and then at 1, 2, 3 and 6 months. In all cases, the samples obtained during therapy were always drawn 2 weeks after the last injection. The blood was collected at room temperature, allowed to clot, centrifuged at 3,000 r.p.m. and then stored at − 20°C until assay. After extraction with diethylether, E2 serum levels were measured by means of radioimmunoassay (RIA), the details of which have been previously published (Trunet et al., 1992). In brief, the RIA uses a specific oestradiol antibody (which has a negligible cross-reaction with E1) and $^{125}$I-labelled oestradiol as tracer. The minimum detectable dose was 3.7 pmol $l^{-1}$, and the within- and between-assay coefficients of variation were respectively 11% and 15%.

Overnight 12 h urine samples (08.00–20.00 h) for 17-OHCS measurements were collected on the day before starting therapy, and at 1, 2, 3 and 6 months. The patients were instructed as to how to collect urine samples, and each of them was given a standard 1 l plastic tube (Kartell, Milan, Italy). In order to check that the patients had collected all of their urine, they were asked to complete a personal card indicating when and how many times it had been collected, especially during the night. On each examination day, the patients had to return the tube and the card (which was checked by nursing staff). Subsequently, the volume of urine was measured and a 20 ml sample was taken and kept frozen (− 20°C) until analysis. Urinary adrenal glucocorticoid metabolite levels were measured by means of gas chromatography (Murphy & West, 1966).

### Treatment plan

The patients were randomised to receive fortnightly i.m. formestane doses of either 250 or 500 mg, injected by nurses. Providing no severe adverse events occurred, the treatment was continued as long as there was no disease progression. At the time of progression, subsequent treatment was given according to the physician's judgement.

### Criteria for tumour response and follow-up

The patients were closely followed, and considered evaluable for response after the administration of at least four doses of formestane.

Objective response (OR) was defined in accordance with UICC criteria (Hayward et al., 1977); complete remission (CR) was defined as the disappearance of all known disease for a minimum of 1 month; partial remission (PR) as a decrease of at least 50% in the sum of the products of the two largest perpendicular diameters of all tumour masses for at least 1 month; stable disease (SD) as a less than 50% decrease or a less than 25% increase in the size of the measurable lesions; progressive disease (PD) as an increase of at least 25% in the size of any tumour lesion or the appearance of new lesions. In this study, the patients classified as having stable disease had to have been stable for at least 12 months. For bone disease, CR was defined as the disappearance during treatment of all completely recalcified lytic bone metastases. In the presence of partial recalcification, the disease was considered as being in partial remission. In no case was pain relief considered an objective response.

In the case of drug discontinuation for progressive disease, the patients were followed up every 2 months in order to record their survival time.

### Statistical methods

The randomisation list (blocks of ten patients for each of the two treatments) was created using Fisher and Yates statistical tables (Fisher & Yates, 1963). It was kept blinded in the Italian Trials in Medical Oncology (ITMO) Data Management Service, and the dose of formestane was disclosed to the physician only at the beginning of treatment in each patient.

The comparison between the baseline E2 levels in the two treatment groups was performed using Student's t-test. The effects of the two fomestane doses on E2 serum and urinary 17-OHCS levels were evaluated using ANOVA, with Dunnett's test being used for multiple comparisons. The Statistical Analysis System (SAS, version 6.04) was used. For E2, the computations were based on the natural logarithm of the original measures in order to achieve normally distributed data. Quantitative data are reported as mean ± standard deviation (s.d.) or standard error of the mean (s.e.m.). A P-value of 0.05 was considered as significant, and 95% confidence intervals were also calculated. The comparison between response rates according to the two different formestane doses was performed by means of the chi-square test.

The duration of response was calculated from the time the best overall response (CR + PR) became evident to the time of progression.

In evaluable patients, the time to progression (TTP) was defined as the period from the date of starting treatment to the date of progression. In all of the randomised patients, the time to treatment failure (TTF) was defined as the period from the date of starting treatment to the date of withdrawal for any cause.

Survival time was defined as the period from the date of starting treatment to the date of death. TTP, TTF and survival time were analysed using the Kaplan–Meier method.

### Results

### Patient characteristics

Between June 1989 and October 1991, 143 consecutive patients were randomised to formestane 250 (72 patients) or 500 mg (71 patients). Six patients were not evaluable for clinical response because they were protocol violators (one patient on 250 mg and two on 500 mg), refused treatment for reasons other than side-effects (two patients on 250 mg) or were lost to follow-up (one patient on 500 mg). Nevertheless, in accordance with the intention-to-treat approach, these six patients were included in the analysis of TTF and overall survival.

Table I shows the main characteristics of the evaluable patients, approximately 60% of whom in each treatment group had a DFI of 2 years or more. It is also worth noting that 12 stage IV patients entered the study. In most of the patients (44 on 250 mg and 37 on 500 mg), spontaneous menopause had lasted for more than 5 years. Fifty-two of the patients (24 on 250 mg and 28 on 500 mg) had not received any previous treatment for advanced disease, while the others had been pretreated with hormonotherapy (41 on 250 mg and 34 on 500 mg) and/or chemotherapy (ten on 250 mg and eight on 500 mg). Twenty-two patients on 250 mg and 15 on 500 mg had had more than one previous treatment for metastatic disease.

*Endocrine effects*

Figures 1 and 2 show the behaviour of serum E2 and urinary 17-OHCS levels in the two treatment groups.

Serum E2 levels were measurable in 131 evaluable patients. At the beginning of the study, the mean levels in the two groups were similar: 21.98 pmol $l^{-1}$ (95% CI 24.14–19.82 pmol $l^{-1}$) in the 250 mg group and 21.40 pmol $l^{-1}$ (95% CI 23.38–19.42 pmol $l^{-1}$) in the 500 mg group ($P = 0.73$, Student's $t$-test). There was no difference in the trend of mean serum E2 levels over time between the two groups ($P = 0.4123$, ANOVA $F$-test for the 'time × group' interaction term), whereas Dunnett's test showed a statistically significant reduction from baseline levels at all treatment assessment times.

Urinary 17-OHCS levels were measurable in 127 patients (64 on 250 mg and 63 on 500 mg). The baseline levels were similar in both groups: respectively 2.05 mg 12 $h^{-1}$ (95% CI, 1.9–2.2 mg 12 $h^{-1}$) and 1.88 mg 12 $h^{-1}$ (95% CI, 1.6–2.1 mg 12 $h^{-1}$). After 6 months of treatment, there was no statistical difference between the urinary 17-OHCS levels of the two groups ($P = 0.4079$), the observed changes falling within the normal range.

*Response to therapy*

Table II shows the response to treatment for each formestane dose. Patients treated with 250 mg achieved a 28% response rate versus 46% for the 500 mg group; this difference was statistically significant ($P = 0.026$). The median duration of response in both groups was 9 months, with a range of 2–40+ months in the 250 mg group and 2–33+ months in the 500 mg group. The median times to CR were respectively 6 and 5 months (5 and 3 months for PRs). The median number of administered doses was 15 in the 250 mg and 18 in the 500 mg group.

Most of the completely responding patients in both groups (about 70%) had a DFI of more than 2 years, and were both ER and PR positive. Three patients in each group had visceral disease. In six patients (three in each group), a very long time of treatment was necessary to achieve tumour regression (an average of 13 months). The median duration of CRs was 11.5 (250 mg) and 13 months (500 mg). Table III shows drug efficacy in relation to disease sites, and it is worth noting the high number of soft-tissue CRs (11/48 on 250 mg and 16/56 on 500 mg). Visceral CRs (five on 250 mg and seven on 500 mg) and bone CRs (three on 250 mg and two on 500 mg) were achieved in both groups; among the visceral lesions, those of the lung were the most responsive in all of the treated patients. Table IV shows the response in relation to major prognostic factors. The response rate was similar in the patients aged more and less than 60 years in the 250 mg group (OR 29%); better results were observed in the over-60-year-olds treated with 500 mg (OR 58% *vs* 34%). In the 500 mg group, better responses were also obtained in patients who were both ER and PR positive (18/31, 58%) or who had a PR level ≥ 50 fmol $mg^{-1}$ protein (14/22, 64%). Four out of ten stage IV patients responded to the 500 mg dose. When formestane was given as first-line treatment for metastatic

**Table I** Main patient characteristics

| | No. of patients 250 mg | 500 mg |
|---|---|---|
| Evaluable | 69 | 68 |
| Median age (range) (years) | 59 (46–75) | 60 (31–71) |
| ER (fmol $mg^{-1}$) | | |
| 10–50 | 24 | 28 |
| >50 | 34 | 27 |
| Unknown | 11 | 13 |
| PR (fmol $mg^{-1}$) | | |
| 25–50 | 10 | 8 |
| >50 | 21 | 22 |
| Negative | 14 | 18 |
| Unknown | 24 | 20 |
| $ER^+PR^+$ | 31 | 32 |
| DFI (years) | | |
| Absent | 2 | 10 |
| <2 | 22 | 18 |
| ≥2 | 45 | 40 |
| Spontaneous menopause | 50 | 44 |
| Oophorectomy | 13 | 16 |
| Drug-induced menopause | 6 | 8 |
| Prior hormonal therapy | | |
| Adjuvant | 12 | 14 |
| For metastatic disease | 41 | 34 |
| Prior chemotherapy | | |
| Adjuvant | 24 | 22 |
| For metastatic disease | 10 | 8 |
| Site of metastatic disease | | |
| Soft tissue | 38 | 41 |
| Viscera | 33 | 25 |
| Bone | 36 | 46 |
| Number of sites | | |
| 1 | 35 | 26 |
| ≥2 | 34 | 42 |

**Figure 1** Mean serum E2 levels (± s.e.m.) by formestane dose. (– ), 250 mg; (—), 500 mg.

**Figure 2** Mean urinary 17-OHCS levels (± s.e.m.) by formestane dose. (– ), 250 mg; (—), 500 mg.

**Table II** Response to treatment

| | No. of patients | |
|---|---|---|
| | 250 mg | 500 mg |
| Evaluable | 69 | 68 |
| Complete remission (%) | 8 (12) | 10 (15) |
| Partial remission | 11 | 21 |
| CR + PR (%) | 19 (28) | 31 (46) |
| Stable disease | 4 | 1 |
| Progressive disease | 46 | 36 |

**Table III** Response related to disease site

| | 250 mg | | 500 mg | |
|---|---|---|---|---|
| Disease site | No. of sites | CR + PR (%) | No. of sites | CR + PR (%) |
| Soft tissue | 48 | 16 (33) | 56 | 32 (57) |
| Skin | 21 | 6 | 23 | 14 |
| Lymph nodes | 22 | 8 | 26 | 14 |
| Breast | 5 | 2 | 7 | 4 |
| Viscera | 46 | 14 (30) | 32 | 10 (31) |
| Liver | 12 | 3 | 12 | 3 |
| Lung | 21 | 6 | 7 | 2 |
| Pleural effusion | 13 | 5 | 13 | 5 |
| Bone | 34 | 6 (18) | 43 | 16 (37) |

**Table IV** Response related to major prognostic factors

| | 250 mg CR + PR | (%) | 500 mg CR + PR | (%) |
|---|---|---|---|---|
| DFI (years) | | | | |
| Absent | – | – | 4 | (40) |
| <2 | 7 | (32) | 5 | (28) |
| ⩾2 | 12 | (27) | 22 | (55) |
| ER (fmol mg$^{-1}$) | | | | |
| 10–50 | 8 | (33) | 12 | (43) |
| >50 | 8 | (24) | 14 | (52) |
| Unknown | 3 | (27) | 5 | (38) |
| PR (fmol mg$^{-1}$) | | | | |
| 25–50 | 1 | (10) | 4 | (50) |
| >50 | 7 | (33) | 14 | (64) |
| Negative | 6 | (43) | 7 | (39) |
| Unknown | 5 | (21) | 6 | (30) |
| ER$^+$ PR$^+$ | 9 | (29) | 18 | (58) |
| No. of disease sites | | | | |
| 1 | 12 | (34) | 14 | (54) |
| ⩾2 | 7 | (21) | 17 | (40) |
| Prior treatment[a] | 11 | (26) | 18 | (45) |
| 4-OHA (first line) | 8 | (31) | 13 | (46) |

[a]For metastatic disease.

disease, the overall response was similar: CRs were obtained in four of the patients on 250 mg (15%), and in six of the patients on 500 mg (21%).

Of the 39 patients in the 250 mg group and the 31 patients in the 500 mg group, who had received TMX for advanced disease, nine (23%) and 17 (55%) respectively responded to formestane. If the responses to formestane are considered in relation to the outcome of previous TMX treatment, the TMX-responsive patients in the 250 mg group responded better than those who were TMX resistant (OR 39% *vs* 13%); the same was true in the 500 mg group (OR 77% *vs* 50%).

Figures 3 and 4 show TTP and overall survival. The median TTP in the two groups was 8 (range 8–46) and 9 months (range 2–35); the median survival time was 30 (range 1–46) and 22 months (range 2–47). No differences in TTF and TTP were observed between the two treatments.

**Figure 3** Time to progression by formestane dose. (–), 500 mg; (---), 250 mg.

**Figure 4** Overall survival by formestane dose. (–), 250 mg; (---), 500 mg.

### *Tolerability*

The local and systemic tolerability of both formestane doses were satisfactory. A few patients complained of mild and transient side-effects; none of them delayed any formestane injection, with the exception of one patient in the 250 mg group who discontinued treatment because of recurrent thrombophlebitis.

Mild asthenia was observed in seven patients in the 250 mg and in six patients in the 500 mg group; one patient in the 250 mg group had grade 3 asthenia requiring concomitant corticosteroid treatment. Hot flushes were reported by two patients in each group; spotting appeared only in three patients in the 250 mg group. Mild nausea and vomiting occurred in four patients in the 250 mg and three patients in the 500 mg group. In two cases (one in each group), grade 2 neutropenia was observed, but the relationship with the drug is uncertain because both of the patients had bone involvement and had also been heavily pretreated with radiotherapy.

Only three patients, two on 250 mg and one on 500 mg, complained of local side-effects consisting of gluteal pain and erythema.

### Discussion

The results of this study of formestane given every 2 weeks to a large number of breast cancer patients confirm our preliminary findings and extend the literature on aromatase inhibitors (Bajetta *et al*., 1992).

The major endocrine effect of formestane has been shown to be a significant reduction in plasma oestrogen levels, mainly E2; unlike AG, the drug causes no change in adrenal synthesis (Samojlik *et al*., 1977, 1978; Coombes *et al*., 1984; Dowsett *et al*., 1987; Lonning *et al*., 1990). In this study, irrespective of the dose, E2 serum levels decreased by an average of 40% from baseline values after only 15 days and remained unchanged thereafter, thus confirming the efficacy of formestane as an aromatase inhibitor. It is interesting to note that the 250 mg dose maintained the maximal decrease in E2 levels throughout the 14 day period between drug administrations. This result is in accordance with the observations of Dowsett *et al.* (1989), although they found that E2 suppression was more variable with 250 mg than with 500 mg and that the phenomenon of recovery occurred prior to reaching steady-state drug conditions. In our opinion, the most important reason for this is that our study population was three times larger.

The action of formestane on the glucocorticoid metabolic pathway has been previously described by Pickles *et al.* (1990), who found that, in patients treated fortnightly with formestane 250 mg i.m., there was a transient fall in serum cortisol levels for 2 weeks, followed by a subsequent increase. The changes remained within the normal daytime range. Our data show that, irrespective of the dose, formestane had no effect on the excretion of 17-OHCS during 6 months of treatment, thus providing biochemical support for the drug's previously reported absence of adrenal toxicity (Coombes *et al.*, 1984; Goss *et al.*, 1986). This finding is accounted for by the high degree of specificity of the drug, which has been documented as inhibiting the aromatase complex without endangering other enzymes (Brodie *et al.*, 1981). The clinical efficacy and the absence of serious side-effects reported in preliminary trials with formestane has led to a number of studies aimed at determining the lowest therapeutic dose, route and scheduling which still achieve maximal E2 suppression. The demonstrable clinical efficacy and low incidence of local side-effects has led to 250 mg i.m. every 2 weeks being selected as the preferred dose (Coombes *et al.*, 1984; Dowsett *et al.*, 1989; Stein *et al.*, 1990; Coombes *et al.*, 1992). To verify these data, we studied the clinical effects of formestane 250 mg and 500 mg.

In our experience, formestane is an effective and well-tolerated endocrine treatment for advanced breast cancer. Although a previous paper has reported that 13% of patients complained of local side-effects (Coombes *et al.*, 1992), we believe that the intramuscular route is well tolerated because such effects were experienced by only three of our patients. Systemic side-effects were also mild and similar in the two groups. The only patient with grade 3 asthenia had liver disease, which probably accounts for the symptom.

Tumour regression was obtained in 28% of the patients treated with 250 mg, and in 46% of those treated with 500 mg. There were no differences between the two doses as far as median duration of response or median TTP, TTF and overall survival are concerned. It is not possible to draw any definitive conclusion concerning the better clinical results obtained with the higher dose, because the size of our study population was calculated in order to detect an endocrinological difference between the two doses and there was no stratification of the main prognostic factors before randomisation. The difference in response rate may be due to a real improvement in drug activity, or to other unpredictable factors. Nevertheless, the evidently greater activity of the higher dose represents an original finding which supports a dose–clinical response effect for formestane and deserves further clinical investigation. In this regard, a dose–biological response effect has already been suggested by the greater inhibition of the peripheral aromatisation of androstenedione into oestrone observed with the 500 mg dose (Jones *et al.*, 1992).

Sequential treatment with formestane after TMX provided satisfactory clinical results. As expected, better results were observed in patients responsive to TMX, although a good response was also achieved in those who were TMX resistant. The responses observed in these latter patients suggest that at least some TMX-resistant tumours remain sensitive to further endocrine therapy, because the failure of TMX may be due to various mechanisms (Johnston *et al.*, 1992).

The 39% overall response of our patients receiving formestane as first-line therapy is interesting. In a previous study, we also found that a major benefit can be obtained in patients who have not received medical treatment before starting formestane therapy (Bajetta *et al.*, 1993). The significant response rate observed with formestane as first-line treatment for metastatic disease supports the choice of formestane in the treatment of patients who have progressed to adjuvant therapy.

In conclusion, the results of the present study involving a large number of patients show no differences between the two formestane doses in terms of endocrine effects, and confirm their efficacy and selectivity. Formestane appears to be very promising in terms of response rate and does not lead to any important local or systemic side-effects. On the basis of these encouraging findings, a multicentre trial involving patients at first relapse, coordinated by the ITMO group, is currently being carried out.

The authors wish to thank the team of data managers from the Italian Trials in Medical Oncology Group (Giuseppa Biasi, Stella Dolci, Rita Finotto, Caterina Somenzi) for their collaboration.

### References

BAJETTA, E., SECRETO, G., ZILEMBO, N., BUZZONI, R., TINESSA, V., MOGLIA, D. & BICHISAO, E. (1992). 4-hydroxyandrostenedione (4-OHA) is effective in postmenopausal advanced breast cancer (ABC) (abstract 38). *Proc. Am. Soc. Clin. Oncol.*, **11**, 53.

BAJETTA, E., ZILEMBO, N., BUZZONI, R., NOBERASCO, C., CELIO, L. & BICHISAO, E. (1993). Efficacy and tolerability of 4-hydroxyandrostenedione (4-OHA) as first-line treatment in postmenopausal patients with breast cancer after adjuvant therapy. *Cancer Treat. Rev.*, **19**, 31–36.

BRODIE, A.M.H., GARRET, W.M., HENDRICKSON, J.R., TSAI MORRIS, C.H., MARCOTTE, P.A. & ROBINSON, C.H. (1981). Inactivation of aromatase in vitro by 4-hydroxyandrostenedione and 4-acetoxyandrostenedione and sustained effects in vivo. *Steroids*, **38**, 693–702.

BRODIE, A.M.H., BANKS, P.K., INKSTER, S.E., DOWSETT, M. & COOMBES, R.C. (1990). Aromatase inhibitors and hormone-dependent cancer. *J. Steroid Biochem. Mol. Biol.*, **37**, 327–333.

COOMBES, R.C., GOSS, P., DOWSETT, M., ETAZET, J.C. & BRODIE, A. (1984). 4-Hydroxyandrostenedione in the treatment of postmenopausal patients with advanced breast cancer. *Lancet*, **ii**, 1237–1239.

COOMBES, R.C., HUGHES, S.W.M. & DOWSETT, M. (1992). 4-Hydroxyandrostenedione: a new treatment for postmenopausal patients with breast cancer. *Eur. J. Cancer*, **28**, 1941–1945.

DEXTER, R.N., FISHMAN, L.M., NEY, R.L. & LIDDLE, G.W. (1967). Inhibition of adrenal corticosteroid synthesis by aminoglutethimide: studies of the mechanism of action. *J. Clin. Endocrinol.*, **27**, 473–480.

DOWSETT, M., GOSS, P.E., POWELS, T.J., HUTCHINSON, G., BRODIE, A.N.H., JEFFCOOTE, S.L. & COOMBES, R.C. (1987). Use of aromatase inhibitor 4-hydroxyandrostenedione in postmenopausal advanced breast cancer: optimization of therapeutic dose and route. *Cancer Res.*, **47**, 1957–1961.

DOWSETT, M., CUNNINGHAM, D.C., STEIN, R.C., EVANS, S., DEHENNIN, L., HEDLEY, A. & COOMBES, R.C. (1989). Dose-related endocrine effects and pharmacokinetics of oral and intramuscular 4-hydroxyandrostenedione in postmenopausal breast cancer patients. *Cancer Res.*, **49**, 1306–1312.

FISHER, R.A. & YATES, F. (1963). *Statistical Tables for Biological, Agricultural and Medical Research*, 6th edn. Edinburgh: Oliver & Boyd.

GOSS, P.E., POWELS, T.J., DOWSETT, M., HUTCHISON, G., BRODIE, A.M.H., ETAZET, J.C. & COOMBES, R.C. (1986). Treatment of advanced postmenopausal breast cancer with an aromatase inhibitor, 4-hydroxyandrostenedione: phase II report. *Cancer Res.*, **46**, 4823–4826.

HAYWARD, J.L., RUBENS, R.D., CARBONE, P.P., HENSOM, J.C., KUMOOKA, S. & SEGALOFF, A. (1977). Assessment of response to therapy in advanced breast cancer. *Br. J. Cancer*, **35**, 292–298.

HOFFKEN, K., YONAT, W., POSSINGER, K., KOLBEL, M., KUNZ, TH., WAGNER, H., BECHER, R., COLLIES, R., FREDERICH, P., WILLMAMS, W., MOOSS, H. & SCHEMIDT, C.G. (1990). Aromatase inhibition with 4-hydroxyandrostenedione in the treatment of postmenopausal patients with advanced breast cancer: a phase II study. *J. Clin. Oncol.*, **8**, 875–880.

JOHANNESSEN, D.C., ADLERCREUTZ, H., FOTSIS, T. & LONNING, P.E. (1993). Plasma and urinary oestrogen in breast cancer patients on treatment with 4-hydroxyandrostenedione. *Br. J. Cancer*, **68**, 393–398.

JOHNSTON, S.R.D., DOWSETT, M. & SMITH, I.E. (1992). Towards a molecular basis for tamoxifen resistance in breast cancer. *Ann. Oncol.*, **3**, 503–511.

JONES, A.L., MACNEILL, F., YACOBS, S., LONNING, P.E., DOWSETT, M. & POWLES, T.J. (1992). The influence of intramuscular 4-hydroxyandrostenedione on peripheral aromatase in breast cancer patients. *Eur. J. Cancer*, **28**, 1712–1716.

LIPTON, A. & SANTEN, R.J. (1974). Medical adrenalectomy using aminoglutethimide and dexamethasone in advanced breast cancer. *Cancer*, **33**, 503–512.

LONNING, P.E. & KVINNSLAND, S. (1988). Mechanisms of action of aminoglutethimide as endocrine therapy of breast cancer. *Drugs*, **35**, 685–710.

LONNING, P.E., DOWSETT, M. & POWELS, T.J. (1990). Post-menopausal estrogen synthesis and metabolism: alteration caused by aromatase inhibitors used for treatment of breast cancer. *J. Steroid Biochem.*, **35**, 355–366.

MURPHY, D. & WEST, H.F. (1966). Urinary 17-hydroxycorticosteroids measured by gas-chromatography. *J. Endocrinol.*, **36**, 331–340.

PICKLES, T., PERRY, L., MURRAY, P. & PLOWMAN, P. (1990). 4-hydroxyandrostenedione-further clinical and extended endocrine observations. *Br. J. Cancer*, **62**, 309–313.

REED, M.J., LAI, L.C., OWEN, A.M., SINGH, A., COLDHAM, N.G., PUROHIT, A., GHILEHIK, N.W., SHAIKN, N.A. & JAMES, V.H.T. (1990). Effect of treatment with 4-hydroxyandrostenedione on the peripheral conversion of androstenedione to oestrone and in vitro tumor aromatase activity in postmenopausal women with breast cancer. *Cancer Res.*, **50**, 193–196.

SANTEN, R.J. (1991). Clinical use of aromatase inhibitors in human breast carcinoma. *J. Steroid Biochem. Mol. Biol.*, **40**, 247–253.

SANTEN, R.J., MANNI, A., HARVEY, H. & REDMOND, C. (1990). Endocrine treatment of breast cancer in women. *Endocrinol. Rev.*, **11**, 221–265.

SAMOJLIK, E. & SANTEN, R.J. (1977). Adrenal suppression with aminoglutethimide. II. Differential effects of aminoglutethimide on plasma androstenedione and oestrogen levels. *J. Clin. Endocrinol. Metab.*, **45**, 480–487.

SAMOJLIK, E. & SANTEN, R.J. (1978). Adrenal suppression with aminoglutethimide. III. Comparison of plasma δ 4- and δ 5-steroids in postmenopausal women treated for breast cancinoma. *J. Clin. Endocrinol. Metab.*, **47**, 717–724.

SCHWARZEL, W.C., KRUGGEL, W. & BRODIE, H.J. (1973). Studies on mechanism of estrogen biosynthesis. VIII. The development of inhibitors of the enzyme system in human placenta. *Endocrinology*, **92**, 866–880.

STEIN, R.C., DOWSETT, M., HEDLEY, A., DAVENPORT, J., ETAZET, J.C., FORD, H.T. & COOMBES, R.C. (1990). Treatment of advanced breast cancer in postmenopausal women with 4-hydroxyandrostenedione. *Cancer Chemother. Pharmacol.*, **26**, 75–78.

TRUNET, P.F., MULLER, P., GIRARD, F., ANPETIT, B., BHATNAGAR, A.S., ZOGRIBI, F., EZZET, F. & MENARD, J. (1992). The effects of fadrozole hydrochloride on aldosterone secretion in healthy male subjects. *J. Clin. Endocrinol. Metab.*, **74**, 571–576.

WORLD HEALTH ORGANIZATION (WHO) (1979). *Handbook for Reporting Results of Cancer Treatment*, WHO Offset Publication No. 48. WHO: Geneva.